# Using homologous network to identify reassortment risk in H5Nx avian influenza viruses

**DOI:** 10.1371/journal.pcbi.1013301

**Published:** 2025-07-22

**Authors:** Ruihao Gong, Zijian Feng, Yanyun Zhang

**Affiliations:** 1 Ministry of Education Key Laboratory for Biodiversity and Ecological Engineering, College of Life Sciences, Beijing Normal University, Beijing, China; 2 School of Systems Science, Beijing Normal University, Beijing, China; Stockholms Universitet, SWEDEN

## Abstract

The resurgence of H5Nx reassortment has caused multiple epidemics resulting in severe disease even death in wild birds and poultry. Assessing H5Nx reassortment risk is crucial for designing targeted interventions and enhancing preparedness efforts to manage H5Nx outbreaks effectively. However, the complexity in H5Nx reassortment, driven by the diversity of influenza A viruses (IAVs) and wide range of hosts, has hindered the effective quantification of reassortment risk. In this study, we utilized a network approach to explore the reassortment history using a large-scale dataset. By inferring genomic homogeneity among IAVs, we constructed an IAVs homologous network with reassortment history embedded within it. We estimated the communities within the IAVs homologous network to represent the reassortment risk of various viruses, revealing diverse reassortment risks across different H5Nx viruses. Our analysis also identified the primary hosts contributing to reassortment: domestic poultry in China, and wild birds in North America and Europe. These primary hosts are critical targets for future H5Nx reassortment interventions. Our study provides a framework for quantifying and ranking H5Nx reassortment risk, contributing to enhanced preparedness and prevention efforts.

## Author summary

As an important evolutionary process, H5Nx reassortment has caused frequent epidemics, resulting in severe disease and even death in various species of wild birds and domestic poultry. Therefore, it is highly important to design effective prevention strategies against future potential H5Nx reassortment. Assessing reassortment risk may be one helpful strategy. However, it is still challenging to evaluate reassortment risk due to the sporadic nature and complexity inherent in the reassortment process. Here, we developed a network-based approach to quantify reassortment risk by collecting all whole genome sequences from the IAVs dataset and constructing an IAVs homologous network embedded with a reassortment history. We then identified network communities to quantify reassortment risk across various viruses and revealed diverse reassortment risks among different H5Nx viruses. By analysing viruses from different hosts, we also identified the primary hosts contributing to reassortment: domestic poultry in China and wild birds in North America and Europe. These primary hosts are critical targets for future H5Nx reassortment interventions. Our study provides a new method for quantifying and ranking reassortment risk in H5Nx, thereby facilitating an effective reassortment surveillance program with a more clearly defined host target.

## Introduction

Influenza A viruses (IAVs) are respiratory pathogens that are characterized by segmented genomes and are composed of eight separate single-stranded RNA segments (PB2, PB1, PA, HA, NP, NA, MP and NS) [1]. H5Nx are subtypes of IAVs classified on the basis of HA (subtypes H1 to H18) and NA (subtypes N1 to N11) [2]. The segmented genome allows H5Nx to evolve by reassortment, such that viral progeny can acquire genomic segments from different parental viruses in coinfected host cells [1]. Coinfection within or between H5Nx may result in intra- or inter-subtype reassortment [3,4]. H5Nx reassortment may facilitate rapid viral evolution, thereby engendering new hosts adaptation and immune evasion [5–9]. Since 2014, the resurgence of reassortment of 2.3.4.4b H5Nx in Eurasia caused frequent cross-species transmission among a wide range of hosts including wild birds and domestic poultry [10–12]. This has resulted in serious epidemics posing severe threats to animal health [13–17]. Therefore, the preparedness for future H5Nx reassortment epidemics is underscored, among which assessing H5Nx reassortment risk may be helpful for tailoring effective prevention strategies.

The diverse genomic compositions of IAVs maintained in a wide range of hosts result in a complex risk for H5Nx reassortment. This diversity is characterized by varied subtypes, diverse internal gene constellations within the defined HA-NA subtypes, and heterogeneity in prevalence [8,18–23]. This heterogeneity means that highly pathogenic avian inﬂuenza (HPAI) mainly endemic in domestic animals, causing severe diseases [24,25], while low pathogenic avian inﬂuenza (LPAI) is more commonly found in migratory birds, exhibiting varied spatial and temporal dynamics [26–28]. IAVs also exhibit high levels of mixed infections in all major hosts [29,30]. Consequently, the diversity of IAVs provides a gene pool for the formation of novel H5Nx variants through inter- and intra-subtype reassortment with complex genome constellations. Additionally, IAVs reassortment occurs sporadically, and different co-infections of IAVs may vary in their compatibility, resulting in varying reassortment potential. This variance introduces complexity to the H5Nx reassortment process [31–34]. The fitness outcomes of reassortment are also uncertain and introduce complexity to IAVs reassortment process [31,35]. Some viruses may become attenuated with reduced fitness, failing to disseminate and eventually expire, while the acquisition of key gene segments may lead to the emergence of more-fit viral strains with enhanced pathogenicity and transmissibility, contributing to reassortment epidemics [36–38].

Furthermore, the susceptibility and exposure among hosts to IAVs adds complexity in H5Nx reassortment risk [20,39]. Hosts with high susceptibility exhibit severe disease and low potential for viral transmission [27]. In contrast, hosts with low susceptibility are able to tolerate multiple viral infections without developing severe disease [40,41]. This facilitates reassortment due to the continuing spread and co-infection of IAVs into susceptible populations [42]. Different hosts vary in their susceptibility due to variations in physiological traits, including immunity, virus binding receptors, behaviour and body conditions [40]. These variations in host susceptibility bring complexity to H5Nx reassortment risk. In addition to host susceptibility, variation in exposure among hosts and environments may influence H5Nx transmission and co-infection, thereby complicating reassortment risk [43,44]. The congregation of wild birds at breeding and wintering sites facilitates H5Nx reassortment among wild bird populations [45–47], whereas the interaction between wild birds and backyard poultry enhances H5Nx transmission and co-infection, thereby increasing the risk of H5Nx reassortment in both poultry and wild bird populations [8,48,49].

The complexity in H5Nx reassortment risk has challenged previous studies aiming at assessing it. Based on in vitro and in vivo experiments, previous studies have been constrained by the partial coverage of complex influencing factors in the study of reassortment risk [50–52]. For example, featured for low immunological susceptibility, the order *Anseriformes* were identified as crucial hosts contributing to H5Nx reassortment [53–55]. However, these studies overlooked the complex cross-species interactions and viral transmission of H5Nx. Furthermore, traditional phylogenetic-based approaches for studying reassortment risk of H5Nx are constrained by the weak handling of large datasets, especially those with complex reassortment histories [56–61]. For example, wild *Anseriformes* were the main hosts in responsible for the 2016/2017 2.3.4.4b H5 reassortment epidemic in Eurasia [60]. However, they are limited to specific epidemics in 2016/2017 with small datasets. Owing to the lack of effective methods, an approach capable of handling the complexity of H5Nx reassortment to quantify and rank the H5Nx reassortment risk is highlighted.

A network-based approach may offer a viable solution for integrating large-scale datasets with complex factors. By inferring the network method, a recent study compiled entire IAVs datasets to detect all reassortant viruses, demonstrating its efficacy in handling large-scale datasets [62]. By incorporating wild animals, livestock, humans, and the complex urban environment, a pathogen transmission network was inferred based on multilayer transmission potential among hosts [63]. This network provides a comprehensive framework for evaluating the role and relative importance of different hosts in facilitating cross-species pathogen transmission. Network analysis is gaining traction as a method with which to translate complex systems into analysable structures [64,65]. In our study, network analysis was inferred on the basis of viral homogeneity to represent reassortment processes with multiple subtypes and hosts involved in evaluating H5Nx reassortment risk.

Herein, we used all IAVs whole genome sequences from the Global Initiative on Sharing All Influenza Data (GISAID) and constructed IAVs homologous networks to represent the H5Nx evolutionary process, including inter- and intra-subtype reassortment. We then estimated the communities of the IAVs homologous networks to determine the reassortment risk. By integrating epidemiological information, we quantified the reassortment risk of various hosts. Our results provide a valuable method for quantifying and ranking the reassortment risk of H5Nx, which may be useful in designing targeted surveillance strategies and enhancing preparedness efforts for future H5Nx reassortment outbreaks.

## Results

### IAVs homologous network construction

**Fig 1:**
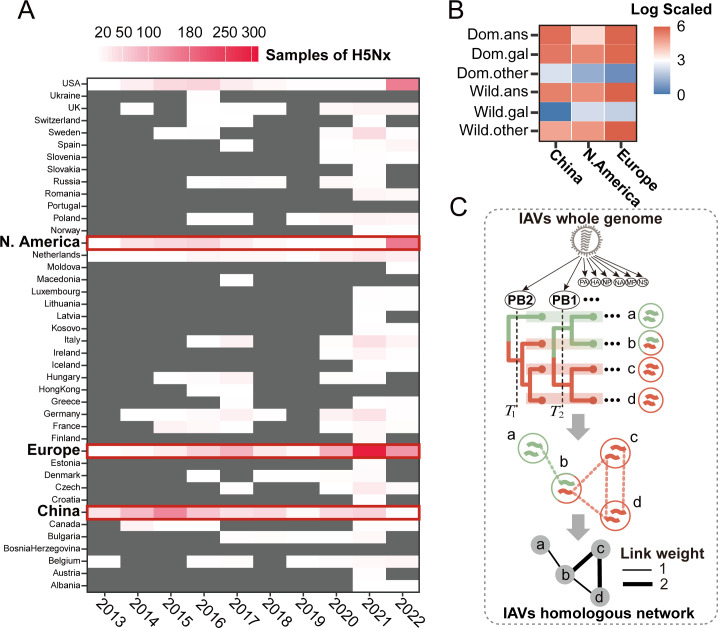
The number of H5Nx whole genome gene sequences and IAVs homologous network construction. (A) Number of whole genome sequences of H5Nx sampled from different regions and countries from 2013 to 2023. The data marked with red borders were key for subsequent analysis. (B) Proportion of different hosts of H5Nx in different regions. (Dom.ans: domestic *Anseriformes*; Dom.gal: domestic *Galliformes*; Dom.other: other poultry; wild.ans: wild *Anseriformes*; wild.gal: wild *Galliformes*; wild.other: other wild birds). (C) The depiction of the IAVs homologous network construction. Whole genome sequences of IAVs were collected to infer maximum likelihood phylogenetic trees for each segment. Segment lineages were partitioned based on median pairwise genetic distance (*T*) among sequences. Each colour represents a specific lineage of that segment. IAVs homologous network were constructed where nodes represent each viral strain, and links formed between viruses with shared segment homogeneity and were sampled within the same year. The number of shared segments serves as the weight for the link.

To represent the H5Nx evolutionary process including inter- and intra-subtype reassortment, IAVs homologous network were first built. A total of 101,214 IAVs whole-genome sequences were collected from the GISAID as of 2023. To mitigate the biases in surveillance intensity across different regions and hosts, the dataset was downsampled in a stratified manner by randomly selecting a varying numbers of sequences per region, host, and lineage (or HA-NA subtype if unavailable), with sampling within one year and over 99% sequence similarity (see Materials and Methods and S1 Table for further details). The final IAVs dataset included a total of 26,031 sequences, with 3420 in H5Nx. The distribution of H5Nx was uneven across spatiotemporal regions and hosts (Fig 1A and 1B). Approximately 24%, 16% and 24% of the sequences were collected from China, North America and Europe, respectively (Fig 1A). Domestic *Anseriformes* hosts (Dom.ans, e.g., domestic duck, goose) and domestic *Galliformes* (Dom.gal, e.g., chicken, turkey, quail) account for 35% and 26% of isolates in China, respectively. Wild *Anseriformes* hosts (wild.ans, e.g., predominantly swans and wild ducks) account for 38% and 26% of isolates in North America and Europe, respectively (Figs 1B and S1).

**Fig 2:**
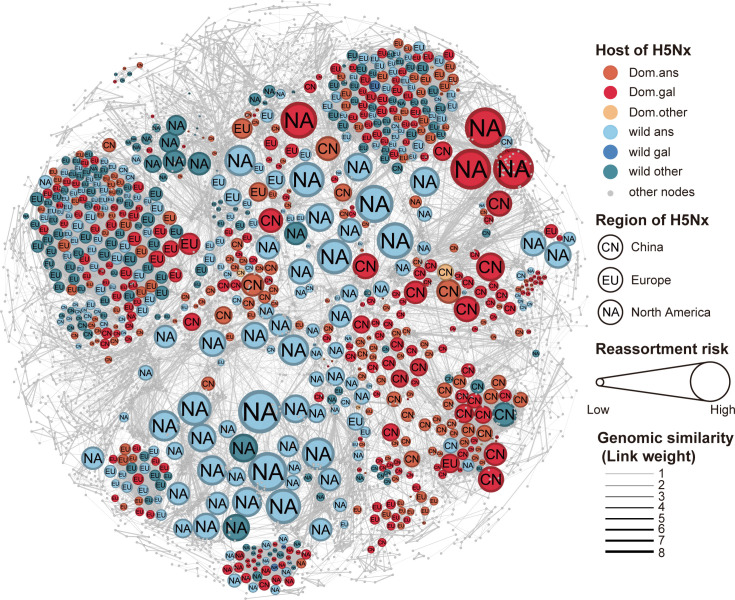
The IAVs homologous network and the H5Nx within it. Each node represents an influenza A virus, and links between viruses represent shared homogeneity, with link thickness representing the number of shared segments (Edge weight). The viral strains of H5Nx, with the abbreviated names of regions, are displayed. Node colours are based on the host type. The names of the regions and host types are shown on the right. The size of each node is proportional to the number of communities (reassortment risk) to which it belongs.

To define the genotype nomenclature of all IAVs, each gene segment from the IAVs dataset was collected to infer the maximum likelihood phylogenetic trees, and the resulting lineages were classified based on median pairwise genetic distance among viruses (S2 Fig). Each lineage of the eight segments and the corresponding epidemiological data, including collection date, location, host, and lineages or subtypes, were combined sequentially (S1 Text). After deduplication based on identical genotype nomenclature, the IAVs homologous network were then constructed, where nodes represent each individual viral strain, and links were formed between two viruses if they shared genomic homogeneity in at least one segment and were collected within the same year. The number of shared segments served as the weight for the link (Fig 1C). The resulting IAVs homologous network consisted of 22,420 nodes and 612,322 links (Fig 2, S1 Dataset).

### The quantification of reassortment risk in H5Nx

To quantify the reassortment risk for H5Nx, the communities within the IAVs homologous network were estimated using the Hierarchical Link Clustering algorithm, identifying a total of 49,758 communities. The community represents collections of nodes with similar genomic compositions, where nodes may belong to multiple communities, representing complex genomic constellations through reassortment with other viruses by acquiring new internal genes. The count of communities to which each virus belongs was measured as an indicator of reassortment risk. We then computed the count of communities for H5Nx viruses, which represents their reassortment risk (Fig 2). The robustness of this method was validated by applying the same strategy in simulated IAVs reassortment phylogenies with varying reassortment rates (S2 Table) and by testing whether viruses that have undergone more reassortment events display higher reassortment risk estimated by the count of communities, as detected by phylogenetic methods (S3 Fig).

### The reassortment risk of different H5Nx host types

**Fig 3:**
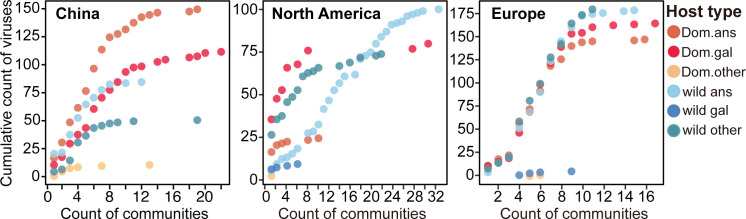
The cumulative distribution of the number of communities across host types and regions in H5Nx. The number of communities represents the number of communities to which each H5Nx virus belongs in the IAVs homologous network and serves as an indicator of reassortment risk. The y-axis represents the cumulative count of viruses with that number of communities or fewer, whereas the x-axis represents the count of communities to which a virus belongs. The colour denotes the host type, with the host name shown on the right.

**Fig 4:**
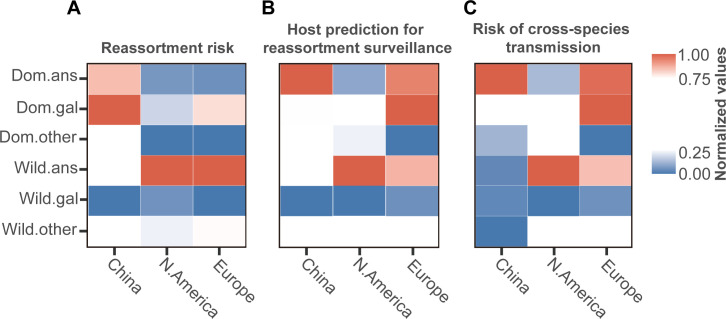
The estimation of key hosts in terms of risk, reassortment surveillance and cross-species segment exchange in H5Nx reassortment. (A) Measurement of the reassortment risk of different hosts in H5Nx. (B) Prediction of key hosts for future surveillance efforts in H5Nx reassortment. (C) Prediction of key hosts in inter-species viral segment movements among different hosts in H5Nx.

To explore the relative risk contributed by different hosts to H5Nx reassortment, we filtered H5Nx viruses collected from 2013 onwards in the IAVs homologous network and counted the number of communities to which each virus belongs. We observed that for different host viruses, the cumulative distribution of the number of communities varied, and the leading host type with the highest community count also differed by region (Fig 3). By computing the average count of communities for each host type across regions, which serves as an indicator of host reassortment risk, we found that in China, poultry *Galliformes* (Dom.gal) have the highest average count of communities, followed closely by poultry *Anseriformes* (Dom.ans). In North America and Europe, wild *Anseriformes* (wild.ans) have the highest average count of communities, followed closely by other wild birds (wild.other) and poultry *Galliformes* (Dom.gal). Consequently, poultry *Galliformes* (Dom.gal) in China and wild *Anseriformes* (wild.ans) in North America and Europe presented the highest reassortment risk, respectively, and serve as key reassortment contributors (Fig 4A). To assess the impact of biased surveillance across different hosts on reassortment risk (Fig 1A and 1B), mutual information measure revealed a non-significant dependency between reassortment risk and host distribution (S3 Table). Finally, to evaluate the applicability of our method, reassortment risk across different hosts in randomly simulated IAVs datasets was estimated to be evenly distributed, confirming the reliability of our method in handling IAVs datasets without introducing false positives (S4 Fig).

### Prediction of high-risk hosts for future surveillance of H5Nx reassortment

Segment exchange due to reassortment leads to connectivity (genomic homogeneity) among viruses and the formation of a network. To evaluate the potential influence of each host type in facilitating reassortment for further surveillance, we assessed how the elimination of connectivity associated with each host type would potentially impact network structure. We found that the removal of connections involving different host types led to different reductions in the number of communities due to the disruption of the original network topology, and the hosts responsible for the most significant reduction in communities also exhibited a high reassortment risk in North America (Fig 4B). However, a subtle difference is observed in China and Europe, where Dom.ans resulted in the most significant reduction in communities in China, while in Europe, Dom.gal was responsible for the most significant reduction in communities followed closely by Dom.ans. This reduction in the count of communities reflects a decrease in reassortment risk associated with host removal, thereby implying a significant potential for viral connection for this host owing to reassortment. Therefore, they serve as potential contributors in facilitating reassortment and thus should be prioritized for surveillance.

To predict the central host facilitating inter-species viral segment exchange, we calculated the reduction in communities associated with non-targeted host nodes. We found that the central host facilitating inter-species viral segment movements is identical to the key hosts under surveillance (Fig 4C). Our results indicated that the key surveillance hosts are also central hosts that promote inter-species viral segment exchange, which underlies cross-species reassortment. Overall, our results suggest that the hosts exhibiting the highest reassortment risk are largely the same as those identified as key hosts for future surveillance efforts.

## Discussion

The reassortment of H5Nx has caused several epidemics throughout history, thus posing significant threats to both domestic poultry and wild birds worldwide. We reconstructed reassortment history by inferring an IAVs homologous network basing on segment genomic homogeneity among IAVs. By estimating network communities, we demonstrated that different viruses in H5Nx exhibited varying reassortment risk. When different host viruses were collected after 2013, Dom.gal in China and wild.ans in North America and Europe presented the highest reassortment risks respectively, both of them followed closely by wild.other and Dom.gal. Although there were subtle differences in China and Europe, the hosts described above also serve as key surveillance targets for effective interventions to prevent future H5Nx reassortment. Further research is needed to elucidate the mechanisms involved in H5Nx reassortment in different hosts.

Numerous reassortment events have been identified across various subtypes and regions [66]. Segment exchange through reassortment among IAVs is prevalent. Therefore, on the basis of IAVs segment homogeneity, we constructed the IAVs homologous network. This network reflects the abundant inter- and intra- subtype reassortment in IAVs due to the connectivity within this network [3,4]; in contrast, several isolated viral groups are present in the absence of reassortment. The reassortment process of IAVs is also embedded within this network.

To assess the underlying reassortment risk, the HLC algorithm was employed to detect the community structure of this network, which represents a collection of viruses with shared genomic homogeneity. However, as a result of genetic exchange, reassortant viruses may harbour segments exhibiting homogeneity with various viruses from different evolutionary backgrounds and may therefore belong to multiple communities [67]. This reflects the reassortment history underlying the viruses and can be inferred to measure the reassortment risk. Owing to complex influencing factors, different viruses in H5Nx may present varying reassortment risks [1,6,40,68].

A wide range of hosts are involved in H5Nx reassortment, including wild waterfowl and avian poultry. Wild waterfowl are natural reservoirs of LPAI IAVs [7,69]. Owing to recent reassortment events of H5 being driven primarily by the extensive reassortment of H5 2.3.4.4 viruses that have emerged since 2013 [10,70], the sequences after 2013 were collected and revealed that for H5Nx, domestic poultry (Dom.gal) exhibited the highest risk in contributing to reassortment in China, followed closely by Dom.ans [71,72]. Along the flyway, migratory birds take multiple stops, which are often surrounded by backyard poultry, thus enabling frequent interactions between wild birds and poultry and facilitating LPAI virus transmission and reassortment [42,49,73–75]. During the past decade, the poultry trade has increased rapidly in China. The density and diversity of species in live poultry markets (LPMs) result in high rates of co-infection and inter-species transmission. The cocirculation of IAVs in LPMs and poultry farms facilitates reassortment [4,76–78]. Furthermore, the weaknesses in biosecurity measures and disease management in Chinese animal agriculture sectors also pose threats to contamination and reassortment in poultry [79–81].

In North America and Europe, wild birds exhibited the highest risk in contributing to reassortment. In North America and European Union countries (EU countries), the poultry industry consists primarily of broiler production systems with limited exposure to wild birds, thus ensuring high biosecurity and reducing the risk of IAVs contamination and mixed infections [82–86]. Along the migration route, high densities of wild birds congregate at breeding and wintering sites, which is associated with high rates of reassortment [45–47,87]. Additionally, in East Asia, the occasional spillover of reassortment from poultry to wild birds can rapidly spread to North America and Europe [14]. However, in Europe, the risk for domestic poultry (Dom.gal) and wild birds (Wild.ans) are ranked closely together. This may be due to the uneven distribution of poultry industry systems and varying biosecurity levels across different countries in Europe, which may increase the risk of reassortment in poultry [25,88].

Finally, we found that the hosts described above are also key targets for future surveillance programs aimed at mitigating the reassortment risk of H5Nx. However, a subtle difference was observed in China and Europe. In China, Dom.ans emerged as the primary host for further surveillance, which may be attributed to the fact that domestic ducks often do not exhibit clinical signs despite shedding the virus, thereby acting as intermediate hosts in the cross-species transmission of avian influenza between domestic poultry and migratory wild birds [49,89,90]. In Europe, Dom.gal was identified as the primary host under surveillance, followed closely by Dom.ans. This may be attributed to the uneven distribution of poultry industry system and varying biosecurity levels in Europe, particularly for Dom.ans, which may serve as intermediate hosts between poultry and wild birds [25,88].

There are some implications in our study. The network-based approach offers a viable solution for integrating large-scale genomic datasets with complex epidemiological factors to quantify reassortment risk, overcoming the constrains of small-scale datasets and the partition coverage of influencing factors in reassortment by traditional methods. This approach provides a more objective and systematic means of quantifying reassortment risk. Our results underscored the need for developing a more targeted host surveillance strategy across regions, particularly focusing on domestic *Galliformes* in China and wild *Anseriformes* in North America and Europe, to increase the efficiency and effectiveness in regular surveillance of avian influenza. Moreover, our results underscore the need to design more targeted biosecurity measures to improve strategies and practices aimed at enhancing biosafety and sanitation measures in Chinese domestic poultry farms and to interrupt cross-species transmission to prevent epidemics. This study sheds light on improvements in preparedness and prevention efforts for future H5Nx reassortment outbreaks.

This study has several limitations. First, despite the downsampling of the IAVs dataset, the limited and biased surveillance of IAVs across time, geography and host species (e.g., according to the FAO and WOAH, only 50% of outbreaks and 0.2% of cases were sequenced) [10] may have biased the construction of an IAVs homologous network. Second, although we categorized wild avian hosts into three groups, migration behaviour can vary among and within bird species, suggesting that these groupings are likely oversimplified. Third, the broad regional categories (e.g., China, North America, Europe) used in sampling location classification may mask important fine-scale geographic variation. Finally, the role of humans in H5Nx reassortment risk is overlooked due to the dead-end host of H5Nx. The role of humans in contributing to H5Nx reassortment may be biased by active medical interventions such as active sampling.

In conclusion, we conducted network analysis to quantify the reassortment risk of different hosts and found that domestic poultry in China and wild birds in North America and Europe present the highest reassortment risk. These primary hosts are also critical targets for future surveillance efforts to enhance the prevention of H5Nx reassortment outbreaks. As reassortment occurs sporadically and continues to pose a threat to both wild and domestic animal health, our quantitative insights into the risk of different hosts in reassortment will benefit the development of more effective prevention strategies for H5Nx reassortment prevention.

## Materials and methods

### Data availability

All available genome sequences of influenza A virus until 2023 were downloaded from the Global Initiative on Sharing All Influenza Data (GISAID) database, with filters excluding duplicated, laboratory derived, environmental sources and other low sequencing quality sequences. To generate a candidate list of viral sequences for further analysis, the sequences were trimmed at the 5′ and 3′ ends to include solely the coding sequence, and sequences with less than 95% completeness of the segment gene length were removed. From these sequence sets, we retained only the complete genome sequences of the influenza viruses. For sequences without collection dates, the midpoint of the corresponding year was used as the estimated sampling date. In total, we obtained a total of 101,214 whole-genome sequences along with epidemiological information, including collection date, clade, host, sampling location and subtypes.

To assess the reassortment risk of different host types among regions, hosts were classified into eight categories based on origin (wild or domestic poultry) and taxonomic order: poultry *Anseriformes* birds (Dom-ans); poultry *Galliformes* birds (Dom-gal); other domestic birds except for *Anseriformes* and *Galliformes* birds (Dom-other); wild *Anseriformes* birds (Wild-ans); wild *Galliformes* birds (Wild-gal); other wild birds except for *Anseriformes* and *Galliformes* birds (Wild-other); and humans and swine. The virus sampling locations were categorized by country and 9 larger region locations, including North America (USA-Canada), Europe, etc. (S4 Table).

To mitigate the biases in surveillance intensity across different regions and host types, we downsampled the sequences in a stratified manner to create a more equitable distribution of IAVs sequences among different hosts. For sequences from over-sampled hosts and regions, a limited number of sequences (at least one) were randomly selected per region, host, and lineage (or HA-NA subtype if unavailable), with sampling within one year and over 99% sequence similarity (estimated using CD-HIT) [91]. For under-sampled hosts and regions, more sequences were randomly selected per region, host, and lineage (or HA-NA subtype if unavailable), following the same temporal and similarity constraints. This strategy increased sampling evenness across host types while retaining a wide range of sampling locations and the overall genetic diversity of the IAVs whole genome dataset.

The original IAVs whole genome dataset (101,214 sequences) was first categorized by subtype into H1Nx (23,960 sequences), H3Nx (56,587 sequences), H4Nx (1820 sequences), H5Nx (8053 sequences), H6Nx (1641 sequences), H7Nx (2794 sequences), H9Nx (1854 sequences), H10Nx (1179 sequences), and other subtypes with fewer than 1000 sequences. For each subtype, sampling locations were either retained at the country level or grouped into larger geographical regions (see S4 Table).

To ensure a more equitable distribution of host types across subtypes, we performed multiple rounds of random downsampling on the original datasets from different regions and subtypes (see S1 Table). Specifically, for H5Nx, sequences were downsampled randomly across hosts and regions as follows:

1) In China, 1 sequence for Dom.ans, 3 sequences for Dom.gal, and 10 sequences for other host types were selected per lineage (or HA-NA subtype if unavailable), with sampling within one year and over 99% sequence similarity, comprising 810 sequences. 2) In North America, 1 sequence for wild.ans and 10 sequences for other host types were selected per lineage (or HA-NA subtype if unavailable), with sampling within one year and over 99% sequence similarity, comprising 557 sequences. 3) In Europe, 1 sequence for Dom.gal, 3 for Dom.ans, 1 for wild.ans, 2 for wild.other, and 10 for other host types were selected per country per lineage (or HA-NA subtype if unavailable), with sampling within one year and over 99% sequence similarity, comprising 804 sequences.

Sampling strategies for other regions in H5Nx are detailed in S1 Table. The final downsampled H5Nx dataset comprises a total of 3420 sequences (S1 Fig).

Similarly, for other regions and subtypes, please refer to S1 Table for the detailed sampling strategies. The final downsampled sequence counts were: H1Nx (7031), H3Nx (8108), H4Nx (1084), H6Nx (1145), H7Nx (1312), H9Nx (1219), H10Nx (654), and other subtypes (2058). By combining all downsampled sequence datasets across subtypes, the final downsampled IAVs dataset comprises a total of 26,031 sequences.

### Genotype nomenclature dataset assembly

We aligned the sequences for each segment in the downsampled IAVs dataset using the MAFFT [92] multiple sequence alignment tool. The aligned sequences were manually edited and cleaned via AliView version 1.26 software [93]. Using these aligned sequences, we inferred a maximum likelihood phylogeny for each gene segment under the GTR + Γ nucleotide substitution model, using randomly selected strains as representatives, implemented in FastTree v2.1.447 [94].

To group clusters of closely related sequences based on their homogeneity for each segment, we first partitioned the evolutionary tree of each segment by a 20th percentile distance threshold using PhyloPart v2.1 [95] to gain a rough division. We then calculated the median pairwise genetic distance among sequences within the same subtype, partition and sampled within one year for each segment. This step required approximately 4 hours using 56 processing threads with hyper-threading. Using this distance as the threshold (S2 Fig), TreeCluster was employed to cluster the sequences in each segment’s evolutionary tree and yield a dataset in which each viral sequence is assigned an index value representing its lineage, with the same index representing sequences belonging to the same lineage [96].

Following the approaches developed by Lu et al. [97], the genotype of the influenza virus was deﬁned as a sequential combination of lineages for each of the eight segments in a genome. Our IAVs genotype nomenclature was defined by the sequential aggregation of the assigned segment index of each segment and epidemiological information. e.g., [PB2, PB1, PA, HA, NP, NA, M, NS, date, host, region, subtype, country]. Genotype nomenclature of all viruses was gathered to create the genotype nomenclature dataset (S1 Text).

### IAVs homologous network construction

By using the genotype nomenclature dataset, viruses with identical genotype nomenclature were deduplicated, retaining only one representative virus. Then IAVs homologous networks were constructed and implemented using a Python program, where nodes represent viruses, and links formed between two nodes if at least one segment shared the same index, which represented genomic homogeneity. Moreover, the collection date interval must fall within a one-year period. The number of shared segments serves as the weight for the link (see S1 Dataset).

### Network community and reassortment risk identification

To estimate reassortment risk, The Hierarchical Link Clustering (HLC) algorithm was used to estimate the community structure of the IAVs homologous network, which required approximately 14 hours to complete. By measuring linkage similarity through sharing neighbour nodes, this algorithm considers a community as a set of closely interrelated links, where each link belongs to a unique community while nodes can participate in multiple communities. This algorithm can find the best community partition threshold by automatically optimizing the partition density (D) value [67]. In the context of our study, the more neighbouring nodes (viruses) are shared between linkages, the closer the relationship between them, thus leading these linkages to be collected into linkage community, where the nodes involved in forming these linkages tend to exhibit greater genomic homogeneity. Therefore, community structure represents a collection of viruses with shared genomic homogeneity. However, as a result of gene exchange, some viruses may share genomic homogeneity with multiple groups of viruses from different evolutionary backgrounds and may therefore participate in multiple communities. The number of communities to which a virus belongs can be used as reassortment risk indicator.

### Reassortment risk of hosts

To estimate the reassortment risk of different host types, H5Nx samples collected after 2013 were extracted because recent reassortment events of H5 were driven primarily by the extensive reassortment of H5 2.3.4.4 viruses that have emerged since 2013. The mean number of communities associated with each host across regions (China, North America, and Europe) was calculated to represent reassortment risk. The risk values were normalized via Min-Max scaling, where the minimum and maximum values in the dataset were mapped to 0 and 1.

To examine whether reassortment risk is dependent on host sampling intensity, we calculated the sample size and corresponding reassortment risk for each host type across different regions. We then computed the mutual information between sample size and reassortment risk, assessing statistical significance through permutation testing with 10,000 random shuffles of the reassortment risk values, thereby generating a null distribution of mutual information scores. The p-value was calculated as the proportion of permuted scores equal to or greater than the observed value. The analysis was performed independently for China, North America, and Europe (S3 Table).

To ensure the applicability of our method without introducing false positive (Type I errors), that is, falsely identify reassortment risk differences when none exist, we randomly generated 500 randomized datasets by independently shuffling the genomic segments, host species, geographic regions, and subtypes in the genotype nomenclature dataset (S1 Text). Subsequently, we applied the same strategy to estimate reassortment risk for each host type within each random dataset and calculated the average risk for each host type (S4 Fig).

### Host surveillance prediction

By referring to the concept of percolation, we assessed how the potential elimination of connectivity associated with each host type would impact network community structure. We removed different host viruses from the IAVs homologous network across regions and recalculated the reassortment risk for each host type in the disrupted network. Owing to connections between nodes of different host types, the removal of viruses belonging to one host may also lead to reductions in communities associated with non-target hosts. The summed difference for all host types between reassortment risk values in the intact and disrupted networks was defined as the reduction in network communities. This reduction reflects a decrease in reassortment risk associated with host removal, indicating the potential of the host in facilitating reassortment. In other words, in the absence of a given host type, reassortment events that would otherwise occur can no longer proceed. The reduction in communities associated with non-targeted hosts indicates cross-species segment exchange between the removed and other non-removed hosts. Hence, the proportion of community reduction associated with non-target hosts was calculated to identify central hosts involved in inter-species viral segment exchange. All sets of values were normalized using Min-Max scaling and ranked to compare host risks, where the minimum and maximum values in the dataset were mapped to 0 and 1.

### Testing the robustness of the methodology by using simulated reassortment phylogenies

To further demonstrate the robustness of our methodology, we simulated whole-genome phylogenies of IAVs with varying reassortment rates (ranging from 0.01 to 0.05) (see explanation in S2 Text) via ARGTools, and detected reassortant and non-reassortant viruses for downstream analysis [98].

To define a simulated influenza nomenclature dataset, we estimated the genetic distance threshold by calculating the median pairwise patristic distances between leaves within the same partitions clustered by PhyloPart v2.1 [95] under a given percentile threshold. Owing to the lack of epidemiological information in the simulated influenza phylogeny, we selected the percentile threshold that produced the same number of partitions as the average number of reassortment events inferred from the simulated phylogeny (see explanation in S2 Text). We then applied this percentile threshold to partition the simulated phylogeny and estimated the corresponding median genetic distance, using the same strategy described in the Methods section, to generate the simulated nomenclature dataset. Using simulated nomenclature datasets, homologous networks were constructed, and the count of communities for each simulated virus was estimated following the same strategy described in the Methods section.

Finally, we tested whether the count of communities for reassortant viruses was significantly greater than that for non-reassortant viruses by using t-tests and Mann-Whitney U tests. This comparison was repeated 10,000 times by randomly selecting an equal number of non-reassortment viruses and comparing them with reassortant viruses. The overall significance (meta p-value) across the 10,000 repeated tests was assessed by applying Fisher’s method to combine the p-values from each test iteration (S2 Table). This method allowed us to validate the consistency of our findings across different datasets and reassortment rates.

### Testing the robustness of the methodology by tracking reassortment process

All H5Nx viruses in the genotype nomenclature dataset were collected and divided into sub-datasets, including H5N2, H5N6, H5N8, China H5Nx, North America H5Nx, and Europe H5Nx. In accordance with the approach described in Bi et al. [99], we downloaded all influenza A virus genomes from GISAID. For each segment in each sub-dataset, BLASTn was performed locally with default parameters against the downloaded sequences. For each virus, the top 100 hits from the BLASTn output were extracted, combined, and deduplicated using SeqKit, resulting in eight sequence datasets corresponding to the eight gene segments of each sub-dataset. Each sequence dataset was then aligned using MAFFT [92]. Maximum likelihood phylogenies were inferred using FastTree with the GTR+GAMMA nucleotide substitution model. The reliability of the inferred phylogenetic splits was assessed using SH-like local support values. On the basis of the phylogenetic topologies obtained and their support values, we classiﬁed them into different lineages according to tree topology and support values of >0.7. According to lineage classiﬁcation of phylogenetic trees, we combined HA, NA and internal genes and designated them different genotypes.

By means of genetic reassortment, the earlier genotype may have diversified into multiple new genotypes due to internal gene replacement. To trace back the reassortment process, we identified parental viruses and later genotypes that shared the backbone of the parental strain but contained one or more replaced segments, which were identified as progeny viruses descended via reassortment.

To test whether the number of communities that reassorted progeny viruses belong to is greater than that of their parental viruses, the differences between the number of communities of each progeny virus and the mean number of communities of its parental viruses for each reassortment process were collected, and a one-sample t test was used to determine whether the mean difference was significantly different from zero (S3 Fig).

## Supporting information

S1 Fig The number of H5Nx viral sequences used in this study before and after downsampling across different host types in China, Europe, and North America. Dark bars indicate the number of sequences before downsampling, while light bars represent the number after downsampling.
(TIF)

S2 Fig Distributions of pairwise genetic distances for each genomic segment of all avian influenza viruses. Kernel density estimates of pairwise genetic distances for each segment (PB2, PB1, PA, HA, NP, NA, MP and NS) are shown in each subplot. Red dashed lines indicate the median distance, and both the mean and median pairwise genetic distances value are shown in each subplot.
(PDF)

S3 Fig Comparison of the number of communities between parental viruses and their progeny descended via reassortment. The Y-axis displays different H5Nx sub-datasets, including H5N2, H5N6, H5N8, China H5Nx, North America H5Nx, and Europe H5Nx and the entire H5Nx. For each dataset, the corresponding boxplots shows the distribution of the differences in the count of communities of each parental virus and their reassorted progeny. The center red line within each boxplot indicates the median value. A one-sample t-test was performed to test whether the difference is significantly greater than zero (indicated by the dashed red line), suggesting that reassorted progeny that have undergone more reassortment events are associated with a significantly higher number of communities compared to their parental viruses. Statistical significance is denoted by red asterisks: *p* < 0.01 (**).
(TIF)

S4 Fig The estimation of reassortment risk of hosts using random simulated datasets. The randomly simulated datasets were generated by shuffling each column of segment indices, collection date, host, location, subtype and countries in the genotype nomenclature dataset (S1 Text). The reassortment risk of hosts were estimated to be evenly distributed.
(TIF)

S1 Table Downsampling strategies for different host types across subtypes and regions. (DOCX)

S2 Table Summary of significance results from random resampling tests comparing reassortant and non-reassortant viruses. (DOCX)

S1 Text The genotype nomenclature dataset. (TXT)

S3 Table Mutual information between sampling rate and reassortment risk of host types across regions, with significance assessed via permutation testing. (DOCX)

S4 Table Regional Classification dataset. The sheet “Regional Classification” lists countries grouped into 9 larger geographic regions based on conventional geographical standards. The sheet “Downsampling regions” defines the regional groupings used for stratified downsampling.
(XLSX)

S2 Text Additional explanation regarding the parameter in S2 Table. (DOCX)

S1 Dataset IAVs homologous network file. (CSV)

S1 File Source data and analysis code. This ZIP archive includes the dataset and code used to reproduce the results of this study.
(ZIP)
