# Supplementary figures and images for: Using homologous network to identify reassortment risk in H5Nx avian influenza viruses
Source: PLoS Comput Biol. 2025 Jul 22;21(7):e1013301. doi: 10.1371/journal.pcbi.1013301 (PMC12282916; doi:10.1371/journal.pcbi.1013301)

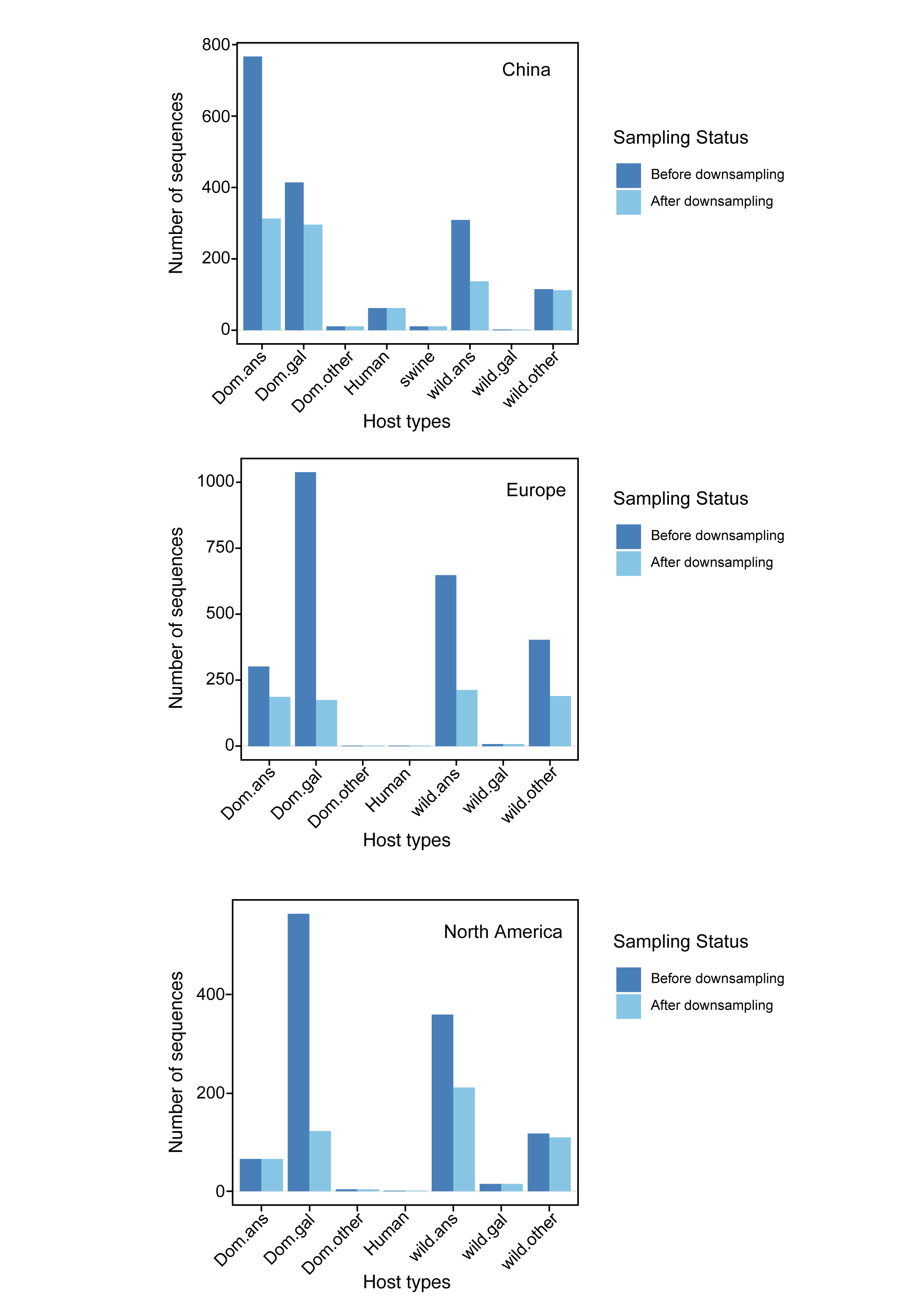

Supplement: S1 Fig — Dark bars indicate the number of sequences before downsampling, while light bars represent the number after downsampling. (TIF) [file pcbi.1013301.s001.tif]

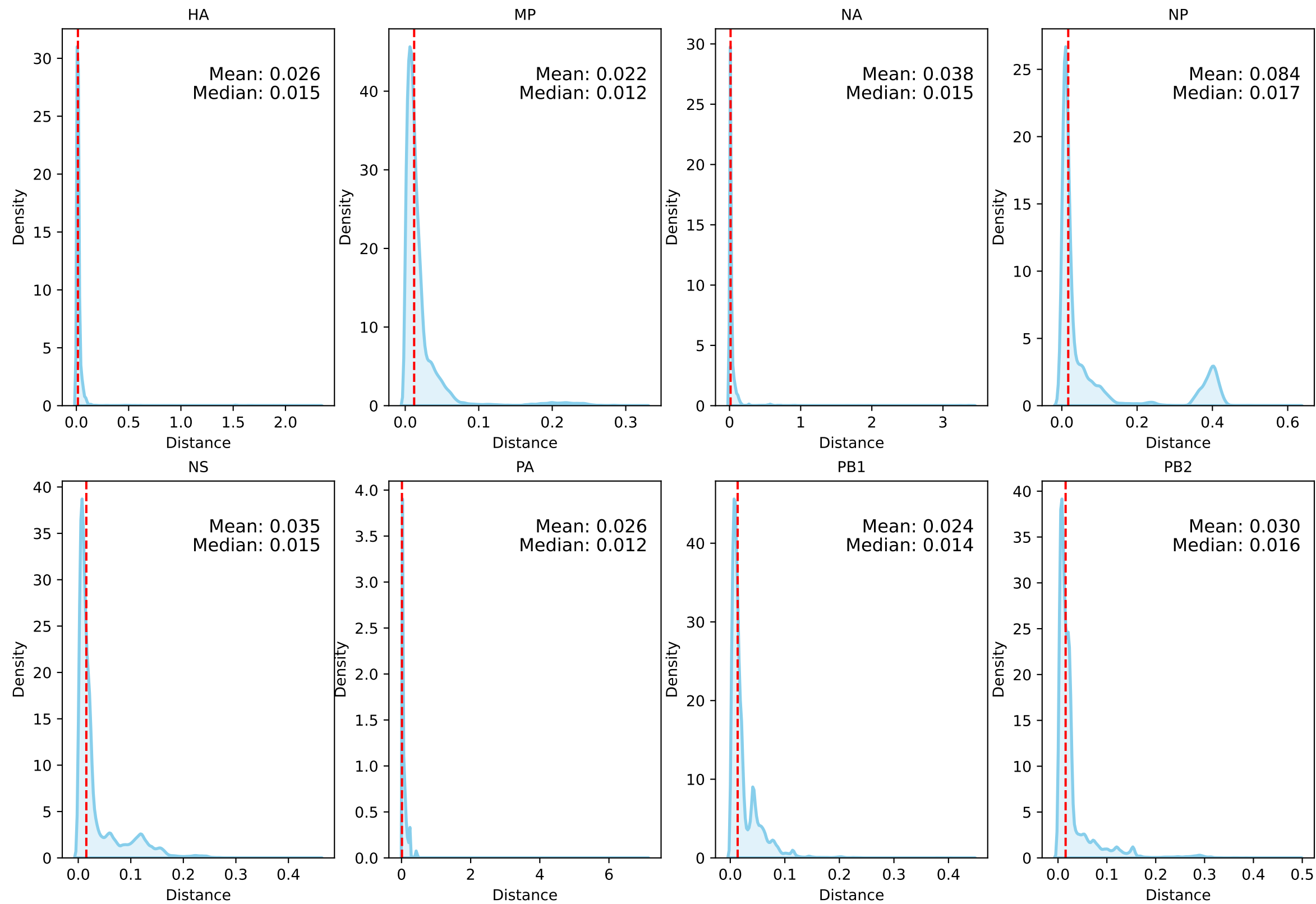

Supplement: S2 Fig — Kernel density estimates of pairwise genetic distances for each segment (PB2, PB1, PA, HA, NP, NA, MP and NS) are shown in each subplot. Red dashed lines indicate the median distance, and both the mean and median pairwise genetic distances value are shown in each subplot. (PDF) [file pcbi.1013301.s002.pdf]

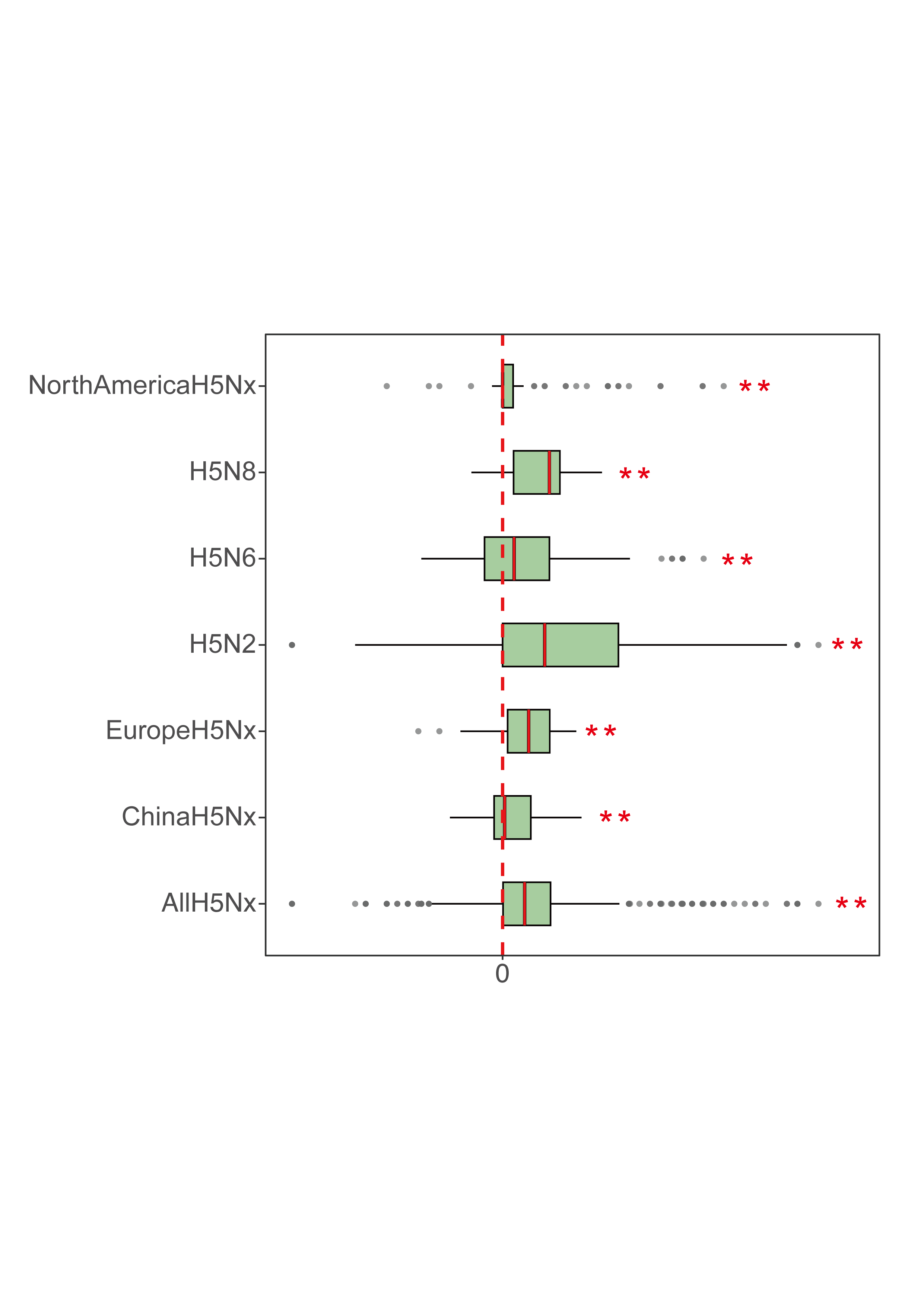

Supplement: S3 Fig — The Y-axis displays different H5Nx sub-datasets, including H5N2, H5N6, H5N8, China H5Nx, North America H5Nx, and Europe H5Nx and the entire H5Nx. For each dataset, the corresponding boxplots shows the distribution of the differences in the count of communities of each parental virus and their reassorted progeny. The center red line within each boxplot indicates the median value. A one-sample t-test was performed to test whether the difference is significantly greater than zero (indicated by the dashed red line), suggesting that reassorted progeny that have undergone more reassortment events are associated with a significantly higher number of communities compared to their parental viruses. Statistical significance is denoted by red asterisks: p < 0.01 (**). (TIF) [file pcbi.1013301.s003.tif]

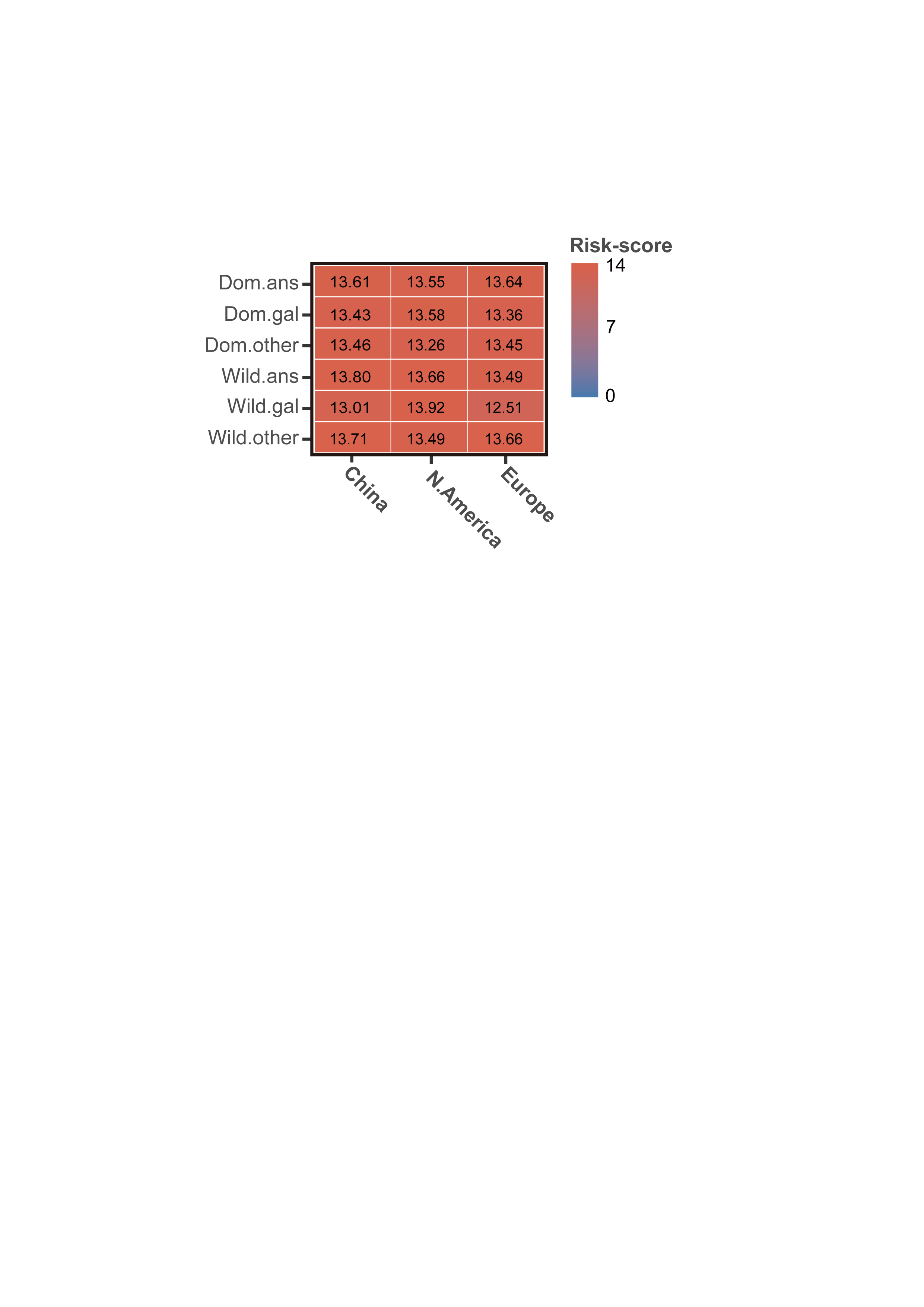

Supplement: S4 Fig — The randomly simulated datasets were generated by shuffling each column of segment indices, collection date, host, location, subtype and countries in the genotype nomenclature dataset (S1 Text). The reassortment risk of hosts were estimated to be evenly distributed. (TIF) [file pcbi.1013301.s004.tif]

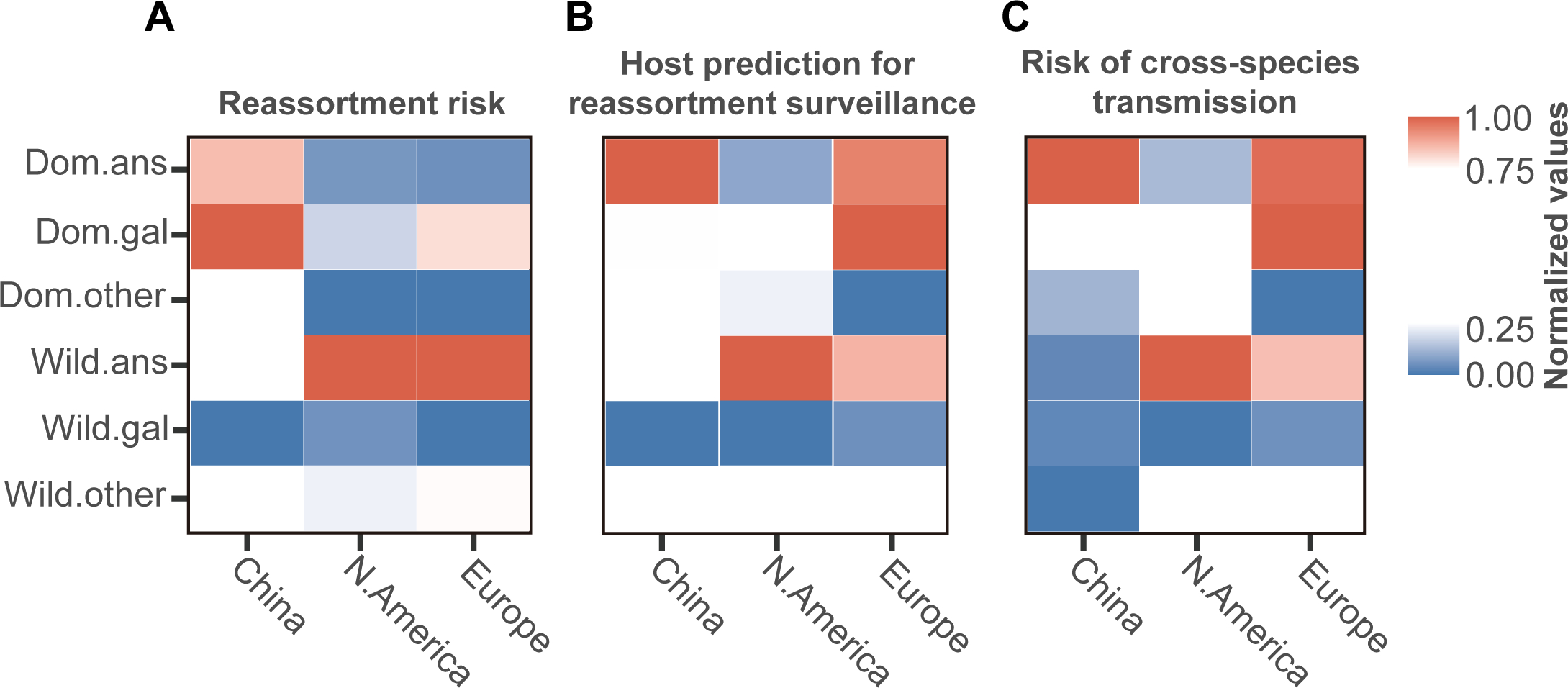

Supplement: S1 File — This ZIP archive includes the dataset and code used to reproduce the results of this study. (ZIP) [file pcbi.1013301.s012.zip › S1_File/step5_Fig4A/Fig4.tif]

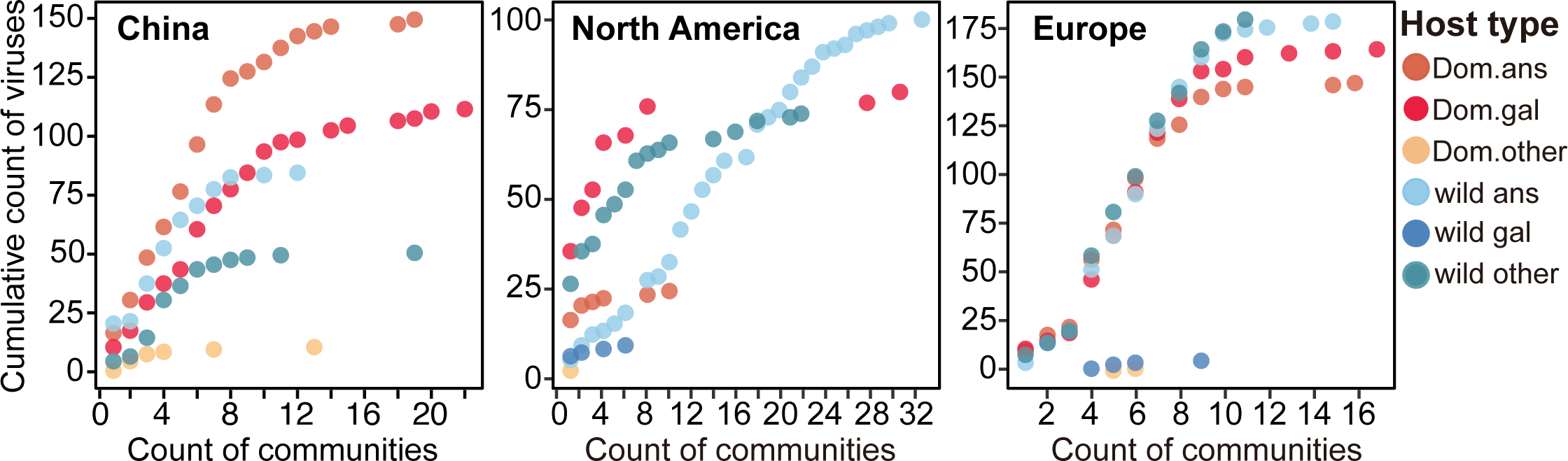

Supplement: S1 File — This ZIP archive includes the dataset and code used to reproduce the results of this study. (ZIP) [file pcbi.1013301.s012.zip › S1_File/Fig3/Fig3.tif]

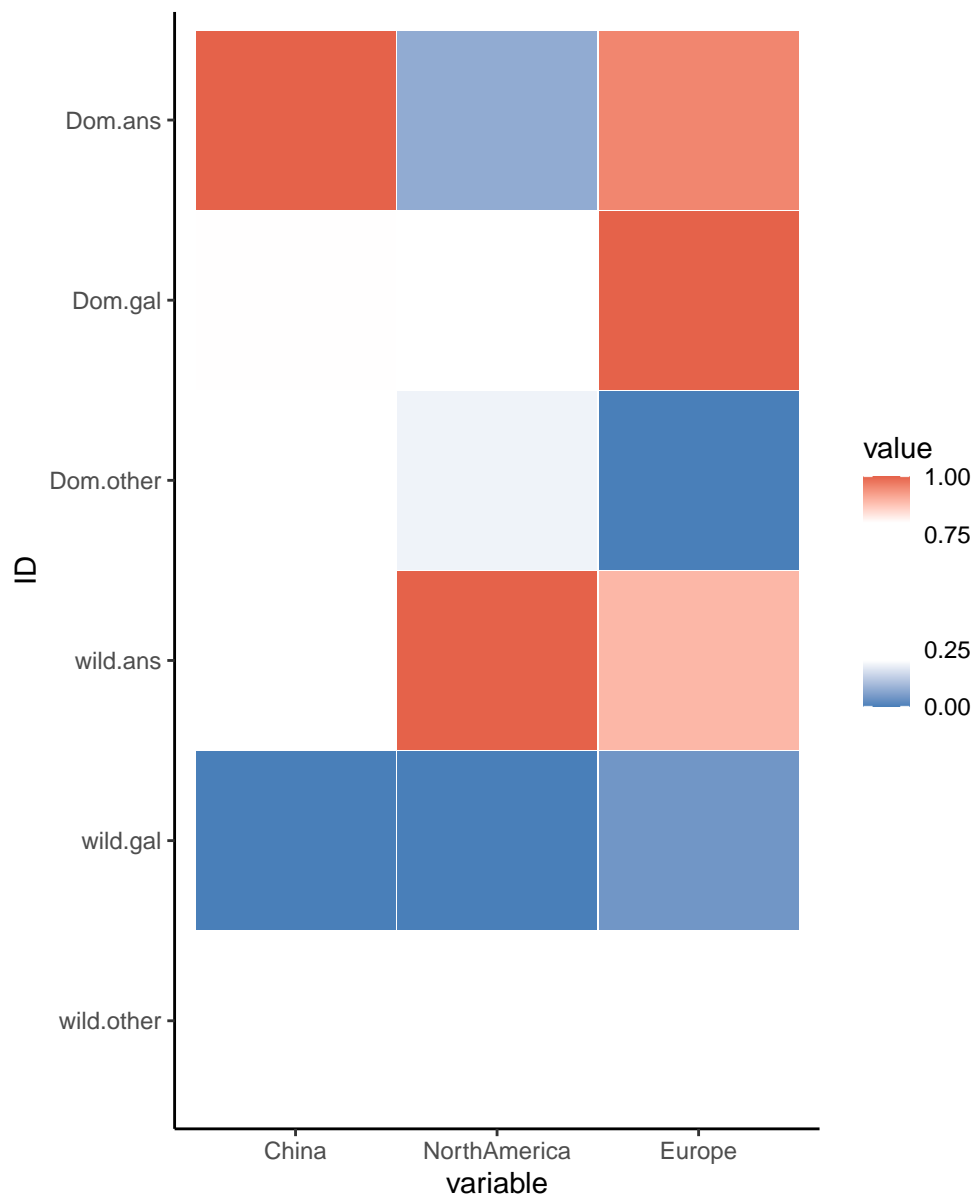

Supplement: S1 File — This ZIP archive includes the dataset and code used to reproduce the results of this study. (ZIP) [file pcbi.1013301.s012.zip › S1_File/step6_Fig4B&C/step3fig4B&C/Host prediction for surveillance.pdf]

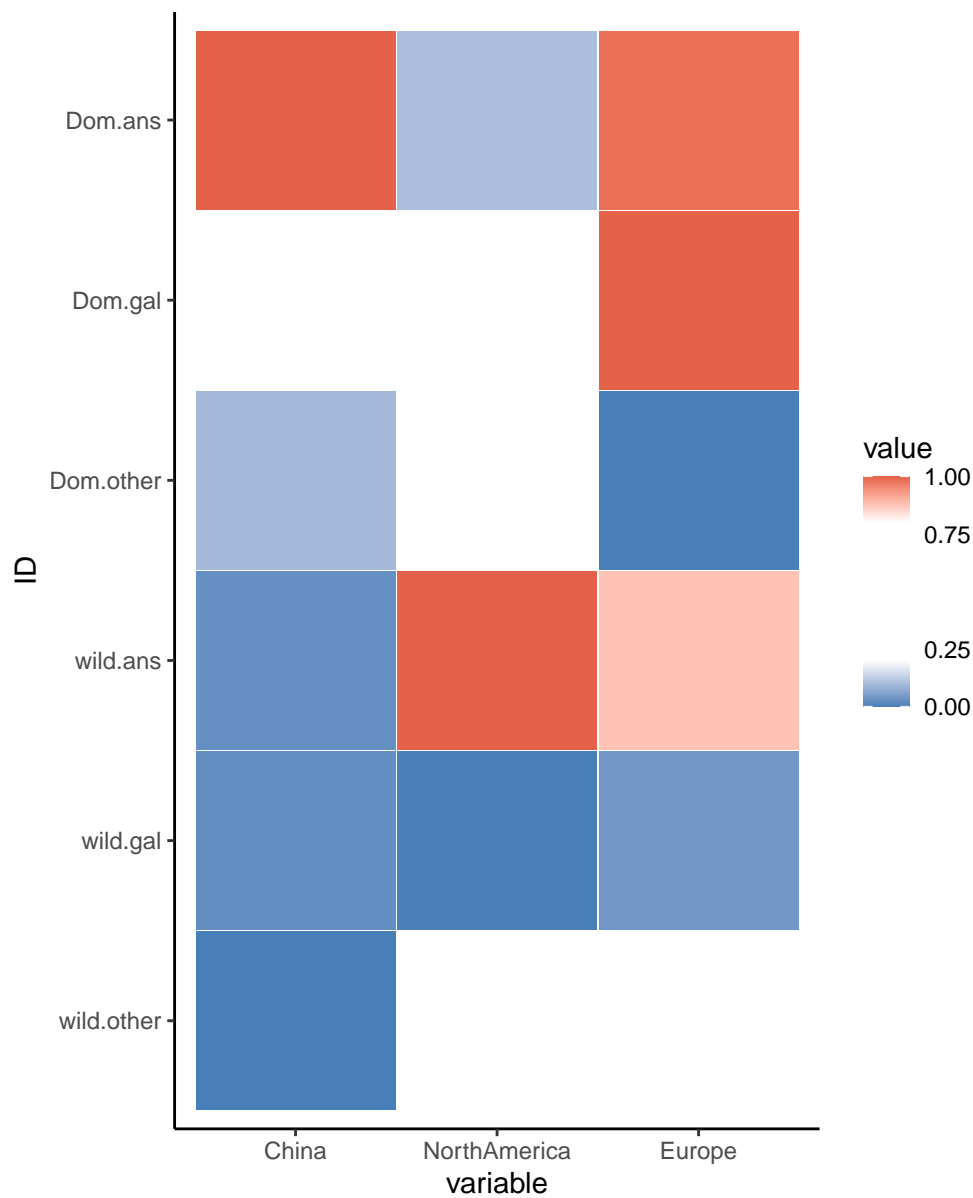

Supplement: S1 File — This ZIP archive includes the dataset and code used to reproduce the results of this study. (ZIP) [file pcbi.1013301.s012.zip › S1_File/step6_Fig4B&C/step3fig4B&C/cross species transmission.pdf]

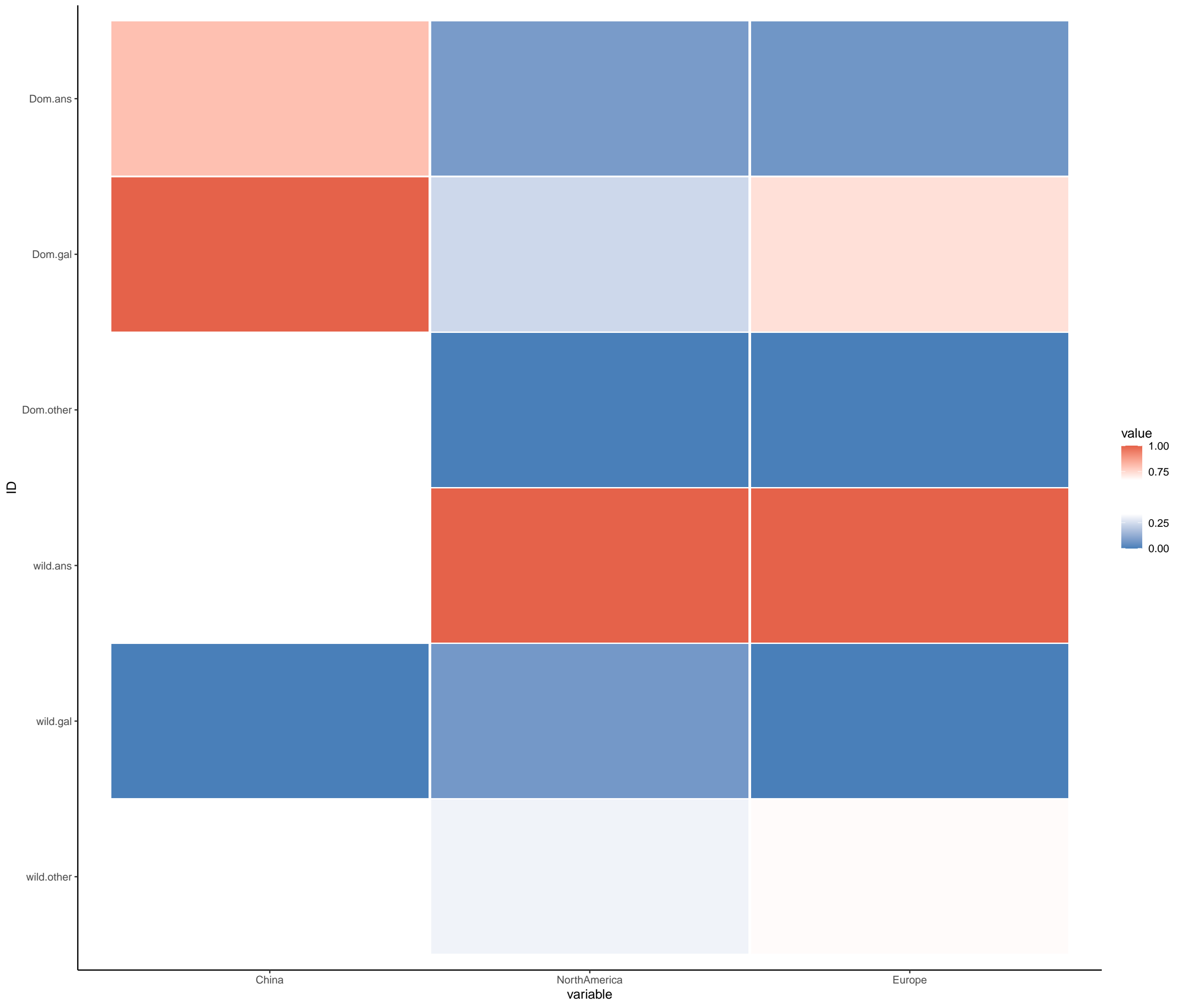

Supplement: S1 File — This ZIP archive includes the dataset and code used to reproduce the results of this study. (ZIP) [file pcbi.1013301.s012.zip › S1_File/step5_Fig4A/fig4A/standardH5.pdf]

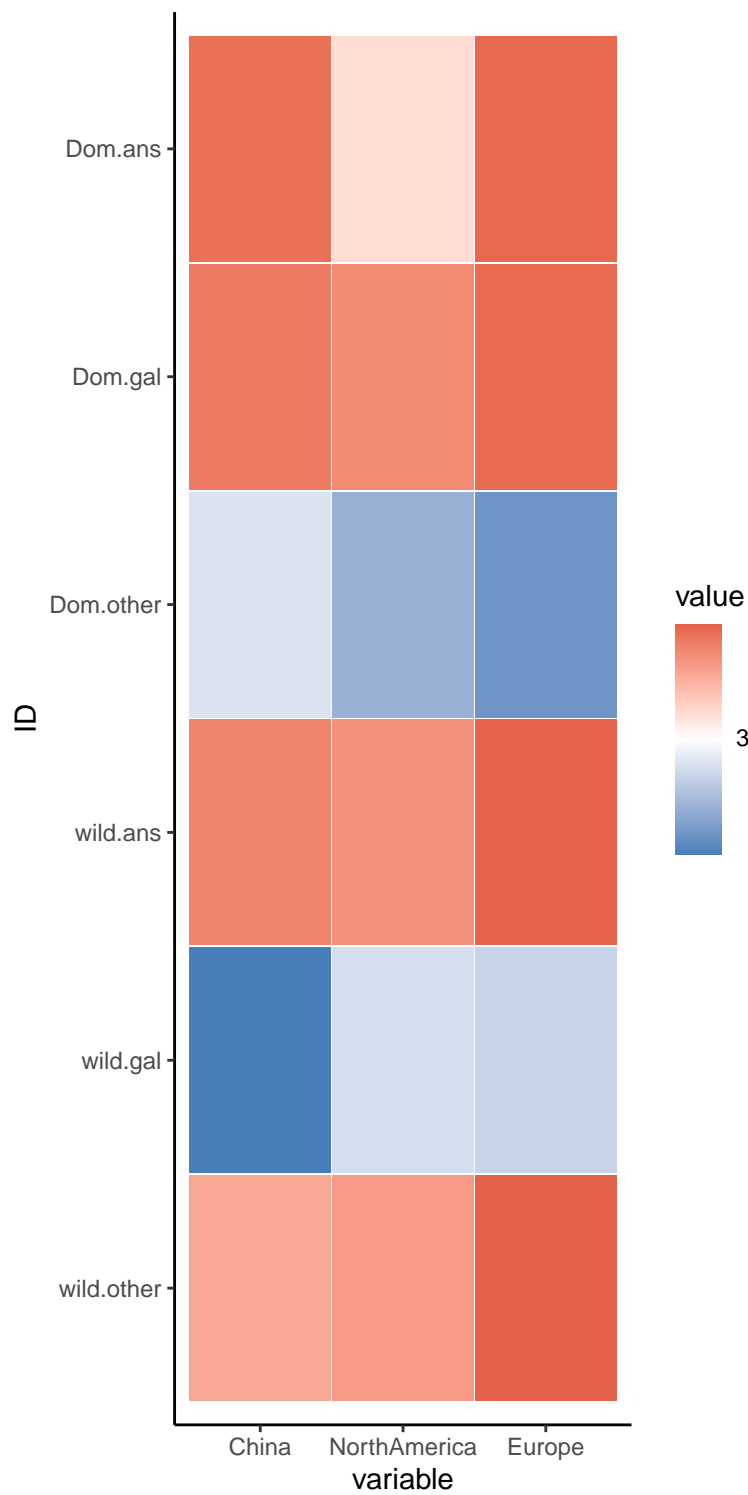

Supplement: S1 File — This ZIP archive includes the dataset and code used to reproduce the results of this study. (ZIP) [file pcbi.1013301.s012.zip › S1_File/Fig1/fig1B/logstandardH5.pdf]

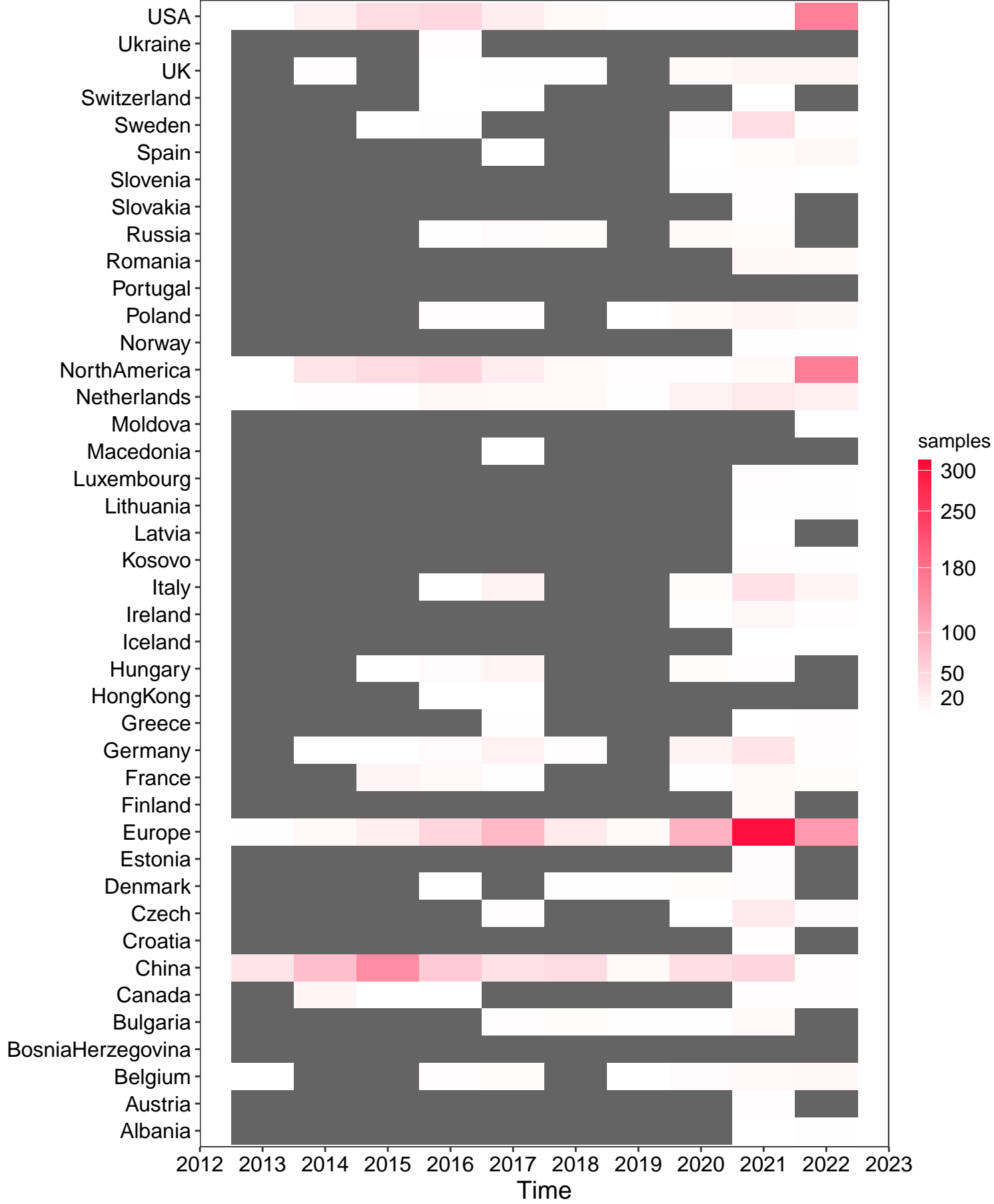

Supplement: S1 File — This ZIP archive includes the dataset and code used to reproduce the results of this study. (ZIP) [file pcbi.1013301.s012.zip › S1_File/Fig1/fig1A/H5_distribution.pdf]
