# Supplementary material for: Using homologous network to identify reassortment risk in H5Nx avian influenza viruses
Source: PLoS Comput Biol. 2025 Jul 22;21(7):e1013301. doi: 10.1371/journal.pcbi.1013301 (PMC12282916; doi:10.1371/journal.pcbi.1013301)
Supplement: S1 Table — (DOCX) [file pcbi.1013301.s005.docx]

S1 Table. Downsampling strategies for different host types across subtypes and regions.

|  | **China** | **North America** | **Europe** | **Africa** | **Latin**  **America** | **South**  **East Asia** | **North Asia** | **Middle East** | **Japan** | **Korea** | **Other** |
| --- | --- | --- | --- | --- | --- | --- | --- | --- | --- | --- | --- |
| **H1Nx**  **(** **23960/7031)** | Swine 1  Other 10 | Swine 1  Other 10 | Swine 1  Human 2  Other 5 | Human 1  Other 5 | Human 1  Other 10 | Human 1  Other 10 | Human 1  Other 10 | Human 1  Other 10 | Swine 1  Human 2  Other 10 | Swine 2  Human 1  Other 10 | Human 1  Other 10 |
| **H3Nx**  **(56587/8108)** | Human 1  Other 10 | Human 3  Other 10 | Human 1  Other 10 | Human 1  Other 10 | Human 1  Other 10 | Human 1  Other 10 | Human 2  Other 10 | Human 1  Other 10 | Human 1  Other 10 | Human 1  Other 10 | Human 1  Other 10 |
| **H4Nx**  **(1820/1084)** | Dom.ans 1  Other 10 | Wild.ans 1  Other 10 | Wild.ans 1  Other 10 | All hosts 1 | All hosts 1 | All hosts 1 | All hosts 1 | All hosts 1 | All hosts 1 | All hosts 1 | All hosts 1 |
| **H5Nx**  **(8053/3420)** | Dom.ans 1  Dom.gal 3  Other 10 | Wild.ans 1  Other 10 | Dom.gal 1  Dom.ans 3  wild.ans 1  wild.other 2  Other 10 | Dom.gal 1  Other 10 | Dom.gal 1  Other 10 | Dom.gal 1  Dom.ans 1  Other 10 | All hosts 1 | All hosts 1 | All hosts 1 | Dom.gal 2  Dom.ans 2  Other 5 | All hosts 1 |
| **H6Nx**  **(1641/1145)** | Dom.ans 1  Other 10 | wild.ans 1  Other 10 | wild.ans 1  Other 10 | All hosts 1 | All hosts 1 | All hosts 1 | All hosts 1 | All hosts 1 | All hosts 1 | All hosts 1 | All hosts 1 |
| **H7Nx**  **(2794/1312)** | Dom.gal 1  Human 1  Other 10 | wild.ans 1  Other 10 | Dom.gal 1  wild.ans 1  Other 10 | All hosts 1 | All hosts 1 | All hosts 1 | All hosts 1 | All hosts 1 | All hosts 1 | All hosts 1 | All hosts 1 |
| **H9Nx**  **(1854/1219)** | Dom.gal 1  Other 10 | wild.other 1  Other 10 | wild.ans 1  Other 10 | Dom.gal 1  Other 10 | All hosts 1 | Dom.gal 1  Other 10 | All hosts 1 | Dom.gal 1  Other 10 | Dom.gal 1  Other 10 | Dom.gal 1  Other 10 | All hosts 1 |
| **H10Nx**  **(1179/654)** | Dom.ans 1  Dom.gal 1  Other 10 | wild.ans 1  wild.other 2  Other 10 | wild.ans 1  Other 10 | All hosts 1 | All hosts 1 | All hosts 1 | All hosts 1 | All hosts 1 | All hosts 1 | All hosts 1 | All hosts 1 |
| **Other subtypes**  **(3326/ 2058)** | All hosts 1 | All hosts 1 | All hosts 1 | All hosts 1 | All hosts 1 | All hosts 1 | All hosts 1 | All hosts 1 | All hosts 1 | All hosts 1 | All hosts 1 |

**Note:**

(1) The number next to each host indicates the maximum number of sequences randomly selected for that host per country/region per lineage (or HA-NA subtype if unavailable), with sampling within one year and over 99% sequence similarity.

(2) “Other” denotes all host types not included among the specifically listed categories in each cell of the table. In this study, host types include Dom.ans, Dom.gal, Dom.other, wild.ans, wild.gal, wild.other, human, and swine.

(3) The numbers next to each subtype in the first column (e.g., 8053/3420) represent the number of sequences before and after downsampling for that subtype.
