# Supplementary material for: Using homologous network to identify reassortment risk in H5Nx avian influenza viruses
Source: PLoS Comput Biol. 2025 Jul 22;21(7):e1013301. doi: 10.1371/journal.pcbi.1013301 (PMC12282916; doi:10.1371/journal.pcbi.1013301)
Supplement: S2 Table — (DOCX) [file pcbi.1013301.s006.docx]

S2 Table. Summary of significance results from random resampling tests comparing reassortant and non-reassortant viruses. For reassortment rates ranging from 0.01 to 0.05, the number of significant results (p ≤ 0.05) across 10,000 resampling iterations is shown for both Mann-Whitney U tests and t-tests. Meta p-values were calculated using Fisher’s method to combine individual p-values across all iterations.

| Reassortment rate | Test | Number of Significance (p < 0.05) | Meta p-value |
| --- | --- | --- | --- |
| 0.01 | U test | 9994 | <0.01 |
|  | t-test | 9998 | <0.01 |
| 0.02 | U test | 2580 | <0.01 |
|  | t-test | 1226 | <0.01 |
| 0.03 | U test | 5152 | <0.01 |
|  | t-test | 1840 | <0.01 |
| 0.04 | U test | 4848 | <0.01 |
|  | t-test | 4039 | <0.01 |
| 0.05 | U test | 9991 | <0.01 |
|  | t-test | 9416 | <0.01 |
