# Supplementary material for: Using homologous network to identify reassortment risk in H5Nx avian influenza viruses
Source: PLoS Comput Biol. 2025 Jul 22;21(7):e1013301. doi: 10.1371/journal.pcbi.1013301 (PMC12282916; doi:10.1371/journal.pcbi.1013301)
Supplement: S3 Table — (DOCX) [file pcbi.1013301.s008.docx]

S3 Table. Mutual information between sampling rate and reassortment risk of host types across regions, with significance assessed via permutation testing.

|  | China | NorthAmerica | Europe |
| --- | --- | --- | --- |
| Mutual Information (MI) | 0 | 0.06 | 0.20 |
| p-value | 1.0 | 0.67 | 0.10 |
