# Supplementary material for: Using homologous network to identify reassortment risk in H5Nx avian influenza viruses
Source: PLoS Comput Biol. 2025 Jul 22;21(7):e1013301. doi: 10.1371/journal.pcbi.1013301 (PMC12282916; doi:10.1371/journal.pcbi.1013301)
Supplement: S2 Text — (DOCX) [file pcbi.1013301.s010.docx]

**Supplementary information**

The file includes:

Additional explanation regarding S2 Table

In main text, to test the robustness of our method in assessing reassortment risk, we randomly simulated influenza whole-genome phylogenies with varying reassortment rates. Then, we constructed simulated homologues network and identified the community structure following the approach described in the Methods section. Finally, we tested whether the count of communities for reassortant viruses was significantly greater than that for non-reassortant viruses (S2 Table).

We would like to append more additional explanation regarding the reassortment rates in simulated phylogenies of IAVs and the genetic distance threshold in defining simulated influenza nomenclature dataset.

**Reassortment rates selection in simulated phylogeny**

We first generated eight simulated phylogenies of whole-genome influenza viral segments using ARGTools [1], setting the reassortment rate to predefined values while keeping all other parameters identical to those in the README example (N = 10,000; n = 5,000; simtype = :flu; r). Here, N is the population size, n represents the number of viruses, and r is the absolute reassortment rate. Then, we identified reassortant and non-reassortant viruses within the simulate phylogeny by applying Treeknit [1].

The Treeknit method works by finding the incompatibilities between two trees to detect reassortment. This is achieved by searching for the maximal sets of leaves that give rise to subtrees with matching topologies from two trees, referred to as Maximally Compatible Clades (MCCs), while the unmatched portions between two trees are interpreted as reassortment.

Hence, a very high reassortment rate leads to reassortment events being detected in nearly every branch (Fig. C), making it difficult to distinguish between reassortant and non-reassortant viruses, as all five nodes may be reassortant in Fig. C. In contrast, a very low reassortment rate results in the sparsity of reassortants (Fig. A). Only an intermediate reassortment rate allows for easier inference of the position of reassortments from the gene trees and accurate differentiation between reassortant (C) and non-reassortant (A, B, D, E) viruses.

**
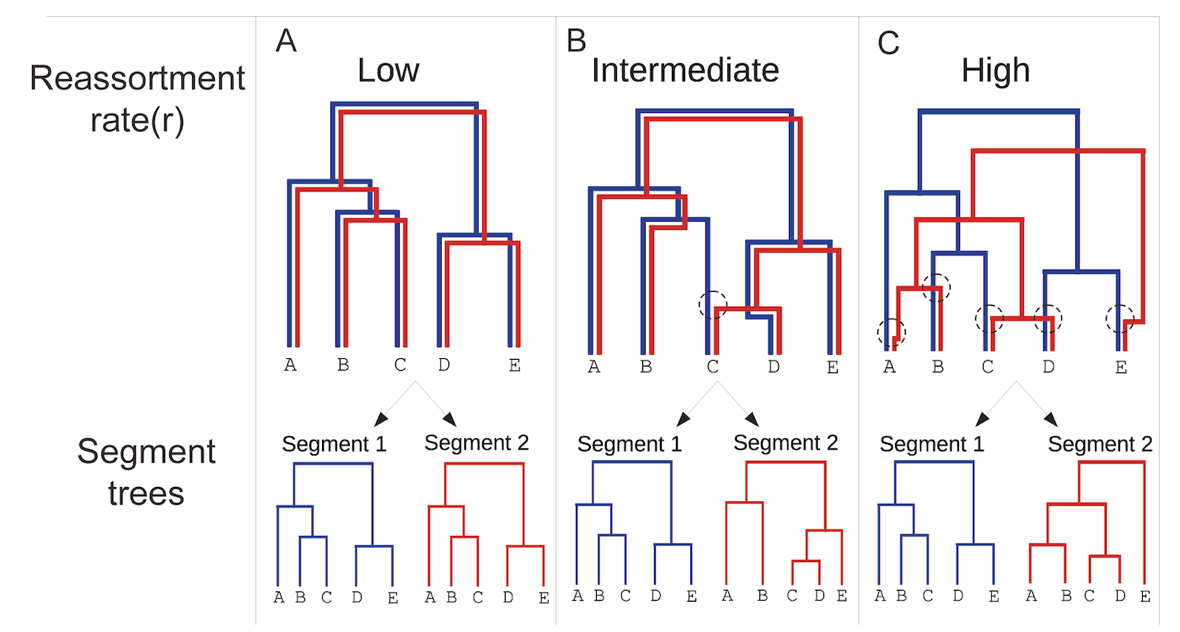
F****igure: Example of reassortment identification in two segment trees using Treeknit.** Reassortments are shown as black circles. (A) Low reassortment rate: reassortments are rare, and the two segment trees are largely compatible with each other. (B) Intermediate reassortment rate: some exchange of segments took place in C through reassortment. Virus C is reassortant, while A, B, D and E are non-reassortant viruses. (C) High reassortment rate: reassortment takes place on every branch, the two segment trees have independent evolutionary histories. **Adapted from Barrat-Charlaix et al. (2022).**

To set a suitable reassortment rate where reassortant and non-reassortant viruses are distinguishable, we experimented with a range of reassortment rates from 0.001 to 0.1. We observed too sparse reassortants at rates lower than 0.01, and too many reassortment events at rates higher than 0.05. Therefore, we selected five reassortment rates ranging from 0.01 to 0.05 to detected reassortant and non-reassortant viruses for downstream analysis. Notable, the rate of 0.05 is close to the actual reassortment rate of A/H3N2 influenza [1].

**Genetic distance thresholds estimation**

To define simulated influenza nomenclature dataset, we set the percentile threshold to produce the same number of partitions in the simulated phylogeny as the average number of reassortment events across all pairs of eight simulated phylogenies. The rationale for this approach is that reassortment introduces new genes, leading to independent evolution into new lineages; therefore, the number of partitions should not be smaller than the number of reassortment events in phylogeny. By applying this percentile threshold to partition the simulated phylogeny by using PhyloPart v2.1 and estimated the corresponding median genetic distance for generating the simulated nomenclature dataset.

**References**

1. Barrat-Charlaix P, Vaughan TG, Neher RA. TreeKnit: Inferring ancestral reassortment graphs of influenza viruses. PLoS Comput Biol. 2022;18(8):e1010394. Epub 20220819. doi: 10.1371/journal.pcbi.1010394. PubMed PMID: 35984845; PubMed Central PMCID: PMCPMC9447925.
